# Combining two national‐scale datasets to map soil properties, the case of available magnesium in England and Wales

**DOI:** 10.1111/ejss.12743

**Published:** 2018-11-23

**Authors:** R. M. Lark, E. L. Ander, M. R. Broadley

**Affiliations:** ^1^ School of Biosciences, University of Nottingham, Sutton Bonington, Nottinghamshire LE12 5RD UK; ^2^ British Geological Survey Keyworth, Nottinghamshire NG12 5GG UK

## Abstract

Given the costs of soil survey it is necessary to make the best use of available datasets, but data that differ with respect to some aspect of the sampling or analytical protocol cannot be combined simply. In this paper we consider a case where two datasets were available on the concentration of plant‐available magnesium in the topsoil. The datasets were the Representative Soil Sampling Scheme (RSSS) and the National Soil Inventory (NSI) of England and Wales. The variable was measured over the same depth interval and with the same laboratory method, but the sample supports were different and so the datasets differ in their variance. We used a multivariate geostatistical model, the linear model of coregionalization (LMCR), to model the joint spatial distribution of the two datasets. The model allowed us to elucidate the effects of the sample support on the two datasets, and to show that there was a strong correlation between the underlying variables. The LMCR allowed us to make spatial predictions of the variable on the RSSS support by cokriging the RSSS data with the NSI data. We used cross‐validation to test the validity of the LMCR and showed how incorporating the NSI data restricted the range of prediction error variances relative to univariate ordinary kriging predictions from the RSSS data alone. The standardized squared prediction errors were computed and the coverage of prediction intervals (i.e. the proportion of sites at which the prediction interval included the observed value of the variable). Both these statistics suggested that the prediction error variances were consistent for the cokriging predictions but not for the ordinary kriging predictions from the simple combination of the RSSS and NSI data, which might be proposed on the basis of their very similar mean values. The LMCR is therefore proposed as a general tool for the combined analysis of different datasets on soil properties.

**Highlights:**

- Differences in sample support mean that two datasets on a soil property cannot be combined simply.
- We showed how a multivariate geostatistical model can be used to elucidate the relationships between two such datasets.
- The same model allows soil properties to be mapped jointly from such data.
- This offers a general basis for combining soil datasets from diverse sources

## Introduction

There are various reasons why national‐scale mapping of soil properties might be required. Policymakers at national and regional scales require a synoptic view of the state of the soil, which may be represented by maps of key indicators (e.g. Robinson *et al*., 2017). Farm advisors or the agricultural industry may benefit from generalized information that shows, for example, where particular problems such as nutrient deficiencies or acidification might be expected to occur (e.g. Lark *et al*., 2014). In this paper we report a study to map available magnesium (Mg) in the topsoil of agricultural land across England and Wales. This was part of a project to examine the risk of Mg deficiency in soil, starting with an overall view of broad national and regional patterns to identify where detailed fieldwork would be undertaken.

In some cases, such maps may be produced from single surveys, such as the National Soil Inventory of England and Wales (McGrath & Loveland, 1992) or the Representative Soil Sampling Survey (Church & Skinner, 1986). Such surveys are costly to undertake, with the total sample size (and hence, the spacing between observations) a major determinant of cost. It would therefore be useful if more than one survey could be combined to provide denser national coverage than either does alone. There is a growing interest in data fusion, the combination of data from multiple sources, to address such problems.

Previous reviews of available soil data for national‐scale soil monitoring in the United Kingdom have identified differences between available datasets, which mean that, *a priori*, the data cannot simply be pooled for purposes of mapping or monitoring (SNIFFER, 2007). In some cases, the soil properties reported are not directly comparable. For example, the data on concentrations of most soil metals in the original National Soil Inventory were obtained by ICP–OES (inductively coupled plasma optical emission spectrometry) on an *aqua regia* extract, whereas data on the same elements in the British Geological Survey's Geochemical Baseline Survey of the Environment (GBase) were determined by XRFS (X‐ray fluorescence spectrometry). Soil pH for the GBase data was measured in calcium chloride, whereas soil pH for the National Soil Inventory in England and Wales was measured in water. Soil properties might, similarly, not be directly comparable between surveys if the sampling date is markedly different and the variable is subject to change over time. Neither are the data directly comparable if the soil is sampled over different depth intervals.

Even when the variables measured in two surveys are directly comparable, it is questionable whether the data can be combined simply if the supports differ. ‘Support’ denotes the size and shape of the volume of soil material that is analysed, to return a single observation in a sample. In the case of the Countryside Survey of Great Britain, the support of the soil data is a single core (Emmett *et al*., 2008), whereas in the case of the GBase survey the support is a physical aggregation of five cores (from depth 0 to 15 cm) collected at the centre and vertices of a 20‐m square (SNIFFER, 2007). When two sets of soil data, obtained by an unbiased sampling design, differ only with respect to their support (i.e factors such as analytical methods or depth interval are identical), then there may be differences in the variance. The process of physical bulking to form a single specimen in the GBase survey is equivalent, at least for simple compositional properties, to an arithmetic averaging and so may be expected to reduce the variance of the resulting data relative to the variance of data where the support is a single core (Lark, 2012).

In this paper we show how one may combine two or more soil datasets for national‐scale mapping by multivariate geostatistical methods. The key idea is to treat one set of measurements as the primary variable (the quantity for which spatial predictions will be made), and the remaining sets as secondary variable(s). This allows us to deal with systematic differences between the primary and secondary variable(s). These include fundamental differences between the variables (e.g. as a result of the analytical method) or differences in support that affect the variance of the observations. The only assumption that is made is that the primary and secondary variables can be regarded as realizations of linearly co‐regionalized random variables (Journel & Huijbregts, 1978). Under these conditions the multivariate geostatistical approach allows us to predict the primary variable with greater precision than we could do by univariate methods applied to the primary data alone. This is because we can exploit the relationship between the primary variable and the secondary variables, even though they cannot be directly combined. The linear model of coregionalization (LMCR) and cokriging have been used most commonly in soil science to facilitate prediction of a target soil variable of interest by cokriging with a scattered covariate, sampled more densely than the target variable (often because it is cheaper and easier to measure). An example is given by Lagacherie *et al*. (2011). The same approach has also been used for the analysis of multitemporal data in studies on soil monitoring (Papritz & Webster, 1995; Lark *et al*., 2006).

We are only aware of one example in the soil literature where the LMCR has been considered as a method to allow the joint analysis of two datasets on the same variable, which cannot be combined for some reason. Rawlins *et al*. (2017) used the LMCR to model the joint spatial variation of two datasets on soil pH in England and Wales; however, their published LMCR parameters do not comprise a valid model and they did not attempt to map the variable with the model. We illustrate this approach using data from two surveys, the Representative Soil Sampling Scheme and the National Soil Inventory, to map the concentration in the topsoil of plant‐available Mg (henceforth, ‘available Mg’) across England and Wales. These two surveys used identical analytical methods on soil sampled from the same depth interval. However, the two surveys use different sample supports. We therefore examine the hypothesis that a consistent model cannot be used for a simple pooling of the two datasets, but that the LMCR offers an approach to model their spatial variation jointly, and to make spatial predictions on the basis of a selected support with meaningful measures of the predictions' uncertainty.

## Methods

### *Surveys and data*

*The Representative Soil Sampling Scheme (RSSS)* The RSSS is described in detail by Church & Skinner (1986). The survey domain was land in agricultural use in England and Wales. The primary sample units were farms. Once a farm was selected for the survey, four fields on the farm were then chosen at random for soil sampling. An aggregated soil sample was formed for each field, comprising 25 cores (depth 0–15 cm). The cores were collected across a 10‐ha subregion of the field or the whole field, whichever was smaller. The data on available Mg were obtained by the procedure of MAFF (1986), which entails extraction with 1 m‐ammonium nitrate.

**Figure 1:**
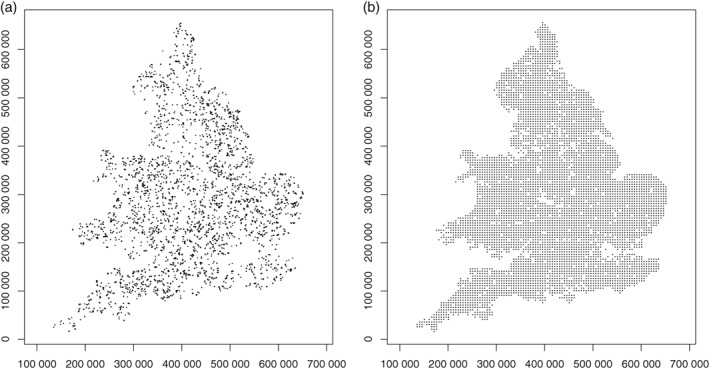
Spatial distribution of data points used from (a) the Representative Soil Sampling Scheme (RSSS) and (b) the National Soil Inventory (NSI). National Soil Inventory Data © Cranfield University (NSRI) and for the Controller of HMSO, 2018. Coordinates are in metres relative to the origin of the British National Grid.

After inclusion in the survey in a given year, a field would then be sampled on two further occasions at 5‐year intervals. The total sample size varied over the period in which the RSSS was undertaken, from 1969 to 2003. In this study we considered only the data from the first sampling of any field. By extracting all such data over the full period of the survey we obtained a total of 8688 observations. We used all these data in an initial analysis to evaluate evidence for temporal trends in the data on available Mg. However, the RSSS did not systematically record unique coordinates for each field until 1981. For subsequent spatial analysis we therefore used data only from fields for which a unique set of coordinates was recorded. This gave a set of 6620 observations. The spatial distribution of these data is shown in Figure 1(a).

*The National Soil Inventory (NSI)* In contrast to the RSSS, the NSI was undertaken as a systematic survey with sample locations at nodes of a 5‐km square grid with origin offset by 1 km north and the same distance east of the origin of the British National Grid (McGrath & Loveland, 1992). At each sample site 25 cores were collected at the nodes of a 5‐m grid in a 20 m × 20 m square, to depth 15 cm. These cores were aggregated to form a bulk sample. The sampling was undertaken between 1978 and 1983. As with the RSSS, available Mg was determined by analysis of an extraction in 1 m‐ammonium nitrate. A total of 5586 data points on available Mg were used from the NSI. The spatial distribution of these data is shown in Figure 1(b).

In summary, these two surveys, after exclusion of RSSS data without unique spatial coordinates, provide similar numbers of observations on available Mg in the soil of England and Wales under agricultural land use. In both surveys the variable was measured on soil to depth 15 cm, and the same extraction method was used. The surveys differ in two respects. First, each RSSS datum represents an aggregated sample over, typically, a 10‐ha area. In contrast, each NSI datum is measured from soil aggregated over 0.04 ha. On this basis we might expect the variance of the RSSS data to be smaller than that of the NSI data, because in the former the effects of variation over a wider range of spatial scales are removed or reduced by the process of aggregation. If the variable is positively skewed, as is often the case for data on nutrients in soil, then differences in support might affect the mean on the original scale of measurement as the mean and variance are not independent. The second difference is the time period over which the samples were collected. The RSSS data we used were collected over 34 years between 1969 and 2003, whereas the NSI data were collected over a 5‐year period. If available Mg is subject to temporal change over this period, then one might subdivide the RSSS data into smaller subsets from more limited time intervals, over which time effects can be regarded as negligible. In a previous study, Baxter *et al*. (2006) undertook spatial analysis of the RSSS data, including available Mg, from four different years (1971, 1981, 1991 and 2001). In the case of available Mg, they concluded that the spatial variation was stable over time, which suggests that one might pool the data. Evidence for temporal variation in available Mg was therefore examined in a preliminary analysis of the RSSS data.

### *Analysis*

#### *Exploratory analysis*

Histograms and summary statistics of both datasets were examined, both on the original scale (mg l^−1^) and after transformation to natural logarithms. Separate summary statistics were also computed for the subset of RSSS data for which unique spatial coordinates were available for each field. A plot of the full RSSS dataset on available Mg against sample date was also examined.

#### *Time effects in RSSS*

We examined the data (transformed to natural logarithms) for evidence of changes in the mean available Mg over time. This was done by an analysis of variance (anova) for differences between sample years. Note that the hierarchical structure of the sampling must be reflected in the anova. There are not 8688 independent observations of available Mg over the years of sampling because farms were selected at random and then four fields within each farm. The anova therefore has two error terms, variation between farms (within sample dates) and variation between fields within farms. It is the residual mean square (RMS) for the former effect against which the RMS for the between‐sample year effect should be tested.

#### *Geostatistical analysis and cokriging*

On the basis of the exploratory analyses it was decided that the RSSS data for Mg could be treated as a single variable. However, the RSSS and NSI datasets differ with respect to their variances. The difference is consistent with an effect of differing sample support. We hypothesize that, as a result of this, the two variables cannot simply be pooled to provide a single dataset for spatial prediction because the pooled dataset cannot be treated as a realization of a stationary random function because of the non‐homogeneity of the variance. We hypothesize that a multivariate treatment of the data, treating them as two variables in a linear model of coregionalization, would allow us to model the data adequately to obtain predictions with meaningful measures of uncertainty. However, this cannot be done with a dataset in which the variables have simply been pooled. Therefore, we undertook a multivariate geostatistical analysis of the RSSS and NSI data on available Mg. The RSSS data, with the smaller variance because of the local aggregation, was treated as the primary variable to be interpolated, using the NSI data as a secondary correlated variable. For comparison we also combined the RSS and NSI data into a single dataset, and undertook spatial analysis with a single variogram of the data used for ordinary kriging. The two models were evaluated using a common set of cross‐validation subsets of data, described below.

We provide only a summary of the multivariate geostatistical method here, and refer the reader to sources where further detail can be obtained (Journel & Huijbregts, 1978; Webster & Oliver, 2007; Chilès & Delfiner, 2012). In summary two variables, *z*_*u*_(**x**) and *z*_*v*_(**x**) measured at locations (**x**), are treated as realizations of two spatially coregionalized random variables, *Z_u_*(**x**) and *Z_v_*(**x**). As in univariate geostatistics, these are assumed to be intrinsically stationary so that the variogram for each variable (auto‐variogram) may be defined, as may the cross‐variogram:

(1) $$\gamma_{u,v}\left(h\right)=\frac{1}{2}E\left[\left\{Z_{u}\left(x\right)-Z_{u}\left(x+h\right)\right\}\left\{Z_{v}\left(x\right)-Z_{v}\left(x+h\right)\right\}\right].$$

There are standard estimators that can be used to obtain estimates of the cross‐ and auto‐variograms from data for specific lags. For the cross‐variogram this is:

(2) $$\begin{array}{cc} {\hat{\gamma }}_{u,v}\left(h\right) & =\frac{1}{2N_{2,1}\left(h\right)}\sum_{i=1}^{N_{2,1}\left(h\right)}\left\{\left(z_{u}\left(x_{i}\right)\right)-z_{u}\left(x_{i}+h\right)\right\}\\ & \left\{z_{v}\left(x_{i}\right)-z_{v}\left(x_{i}+h\right)\right\}, \end{array}$$

where *N*_2, 1_(**h**) pairs of observations of *z*_1_ and *z*_2_ are separated by the lag interval **h**. If predictions of one or both variables are to be obtained by cokriging then a model must be fitted to the estimates. In practice, the most common approach is to fit a linear model of coregionalization (LMCR). In an LMCR two or more variables are treated as the linear combination of a common set of independent random variables, denoted by $y_{j}^{k}\left(x\right)$, where *k* is an index, not a power. The standardized variogram function *g* ^*k*^(**x**) is common to all variables in this set with the same index *k*. In this study we used simple models with a spatially uncorrelated component (*k* = 1) and a single spatially correlated component (*k* = 2), although more complex models, such as the double spherical, with a second spatially correlated component could also be fitted. Under the LMCR the random variable *Z*_*u*_ is then given by:

(3) $$Z_{u}\left(x\right)=\sum_{k=1}^{2}\sum_{j=1}^{2}a_{u,j}^{k}\;y_{j}^{k}\left(x\right),$$

and a similar expression with terms $a_{v,j}^{k}$ defines *Z*_*v*_. The auto‐ and cross‐variograms for *Z*_*u*_ and *Z*_*v*_ can then be written as:

(4) $$\begin{array}{c} \gamma_{u,u}\left(x\right)=b_{u,u}^{1}+b_{u,u}^{2}g^{2}\left(x\right)\\ \gamma_{v,v}\left(x\right)=b_{v,v}^{1}+b_{v,v}^{2}g^{2}\left(x\right)\\ \;\gamma_{v,u}\left(x\right)=b_{v,u}^{1}+b_{v,u}^{2}g^{2}\left(x\right), \end{array}$$

where

(5) $$b_{u,v}^{k}=\sum_{j=1}^{2}a_{u,j}^{k}a_{v,j}^{k}.$$

The coregionalization matrices,

$$\left[\begin{array}{ll} b_{u,u}^{k}, & b_{u,v}^{k}\\ b_{u,v}^{k}, & b_{v,v}^{k} \end{array}\right],$$

are constrained to be positive definite in the LMCR. If one standardizes the corregionalization matrices to correlation matrices in the usual way, then the off‐diagonal term for each matrix is called the structural correlation between the variables at the scale represented by the variogram function *g* ^*k*^(**h**) (Goovaerts & Webster, 1994).

We are proposing the LMCR as a model for the joint analysis of data from two soil surveys. In such a case, the observations are not, in general, available at coincident sites, which means the standard estimator of the cross‐variogram in Equation (2) cannot be applied. In such cases, the pseudo‐cross variogram, $\gamma_{2,1}^{P}\left(h\right)$, of Myers (1991) based on a proposal of Clark *et al*. (1989), may be used:

(6) $$\gamma_{2,1}^{P}\left(h\right)=\frac{1}{2}\mathrm{var}\left[Z_{2}\left(x+h\right)-Z_{1}\left(x\right)\right],$$

where var[·] denotes the variance of the term in brackets.

A comprehensive account of the pseudo cross‐variogram is given by Papritz *et al*. (1993), and we do not go into further detail here, only making the observation that it does not exist in general for processes that are only intrinsically stationary but can be defined for second‐order stationary processes for which the variogram model approaches or reaches a bounding value with increasing lag.

Papritz *et al*. (1993) propose a general centred estimator of the pseudo cross‐variogram ${\hat{\gamma }}_{u,v}^{P,\mathrm{Pa}}$:

(7) $${\hat{\gamma }}_{u,v}^{P,\mathrm{Pa}}\left(h\right)=\frac{1}{2N_{2,1}\left(h\right)}\sum_{i=1}^{N_{2,1}\left(h\right)}{\left\{\left[z_{u}\left(x_{i}+h\right)-{\bar{z}}_{u}\right]-\left[z_{v}\left(x_{i}\right)-{\bar{z}}_{v}\right]\right\}}^{2},$$

where *N*_*u*, *v*_(**h**) pairs of observations of *z*_*u*_ and *z*_*v*_ are separated by the lag interval **h**. The arithmetic means of the two datasets are denoted by ${\bar{z}}_{u}$ and ${\bar{z}}_{v}$.

We estimated the pseudo cross‐variogram for the data on available Mg from the NSI and RSSS, as well as their separate auto‐variograms. Exploration of the data showed no evidence of pronounced anisotropy (directional dependence), and so we estimated variograms for scalar lag distances, *h*, rather than lag vectors, **h**. We then fitted parameters of the LMCR by weighted least squares, using the simulated annealing algorithm of Lark & Papritz (2003). As noted by Papritz *et al*. (1993), fitting this model when no estimate of the pseudo‐cross variogram is available for lag zero requires some assumptions, and we followed Lark (2002) in making the conservative assumption that the covariance term in the nugget coregionalization matrix, $b_{u,v}^{1}$ was zero.

When an LMCR has been fitted for two or more variables, then spatial predictions of one of them (the primary variable) may be computed as linear combinations of the data on all variables. The weights are found that minimize the expected squared prediction error, or cokriging variance. Ordinary cokriging was used to compute predictions of available Mg using the RSSS data as the primary variable and NSI data as the secondary variable. Predictions were obtained at the nodes of a 500‐m square grid. Ordinary kriging from the RSSS data alone, or from the pooled RSSS and NSI data (variogram for the pooled data), was undertaken using the cokb3d program from the GSLIB library of Fortran geostatistical code (Deutsch & Journel, 1998).

As a test of the LMCR and cokriging procedure, and for comparisons with ordinary kriging using only the RSSS data, or the RSSS and NSI data pooled into a single set, a cross‐validation was undertaken. Fifty farms in the RSSS survey were selected independently and at random and removed from the available set for kriging. A validation set was produced by selecting one field independently and at random from each of the 50 farms. The values at the 50 locations in this validation set were computed by cokriging from the remaining data, including all the NSI available Mg data. The same values were also predicted by (i) ordinary univariate kriging from the remaining RSSS data alone and (ii) ordinary kriging from the remaining RSSS data combined with the NSI data, using the variogram estimated from this pooled dataset. This was repeated ten times over, with no farm included in more than one validation set. A total of 500 cokriging predictions and corresponding univariate kriging predictions were therefore obtained with the corresponding measured values and the cokriging and kriging variances of the predictions. We computed the mean cokriging and kriging errors; however, the main test of the cokriging and univariate ordinary kriging predictions and their associated models was undertaken by computing the standardized squared prediction errors (Lark, 2000) at each of the validation sites:

(8) $$\theta \left(x_{0}\right)=\frac{{\left\{\tilde{Z}\left(x_{0}\right)-z\left(x_{0}\right)\right\}}^{2}}{\sigma_{\mathrm{CK}}^{2}\left(x_{0}\right)}.$$

This statistic tests the validity of the kriging variances computed for the cokriging predictions. The equivalent statistic for the univariate ordinary kriging predictions was also obtained. The expected value of this statistic is one, but, as noted by Lark (2000), this may be influenced by outlying values and the best test of the predictions is the median value, which has an expected value of 0.45 in the case of normal kriging errors with an unbiased kriging variance. The median value of the standardized squared prediction error was computed over all 500 cross‐validation sites. Approximate confidence limits for the median value of *θ* with an unbiased kriging variance were obtained, assuming that the cokriging errors are independent random variables and using the conventional normal approximation for the sample median (Freund, 1992). The cross‐validation outputs were used to test and compare the validity of the kriging or cokriging variances by computing the coverage of prediction intervals. For any cross‐validation prediction, a prediction interval may be computed about the prediction for a specified probability, assuming a normally distributed error, and standard error equal to the square root of the kriging variance. One may then note for each prediction whether the prediction interval includes the observed value. Over an independent random sample the proportion of observations where the prediction interval includes the observation, which is called the coverage of the interval, is expected to equal the expected probability.

Coverages were estimated for probabilities from 0.5 to 0.99 for each of the three sets of cross‐validation predictions. The 95% confidence interval for each estimated coverage was computed with the blakerci function in the PropCIs package for the R platform (Scherer, 2018), which obtains the exact confidence interval for a binomial proportion according to the method of Blaker (2000).

## Results

### *Exploratory analyses*

**Figure 2:**
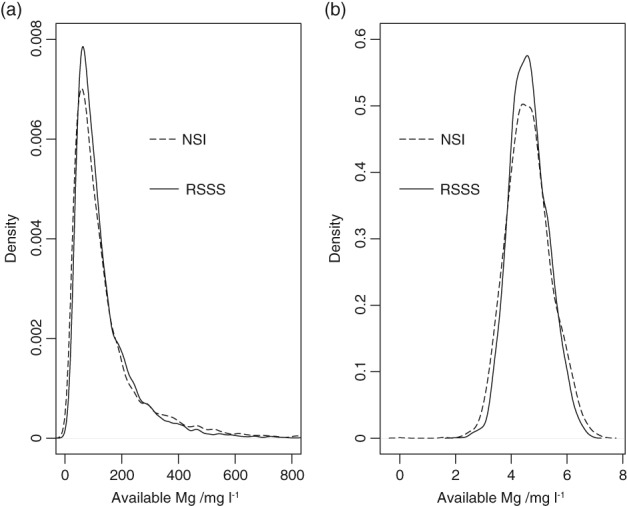
Empirical density functions for concentration in the topsoil of plant‐available Mg in NSI and RSSS soils on (a) original scale of measurement and (b) after transformation to natural logarithms. Note that the abscissa of Figure 2(a) has been truncated. The densities for both datasets are very small between the maximum value shown and the maximum value in the dataset (1601 mg l^−1^).

Summary statistics for the separate datasets are shown in Table 1. There is little difference between the statistics for the full RSSS dataset and the reduced set (from which farms were removed if all fields had a single set of coordinates recorded). All datasets had a large skewness coefficient, which was reduced to a small value on transformation to natural logarithms. Note that the mean and median values for the NSI data and the RSSS data on the log scale are close. The standard deviation of the NSI data is somewhat larger. Figure 2 shows the empirical density plots for the NSI data and reduced RSSS dataset on the original and transformed scales. These plots were produced with the density procedure for the R platform (R Core Team, 2017). Note the pronounced positive skewness of both variables on the original scale of measurement, and the more symmetrical distribution on the log scale. The NSI data have markedly heavier tails to their distribution, which is consistent with the larger variance. This is the expected consequence of the difference in support between the two datasets. The NSI data are aggregated over a small (0.04 ha) plot, whereas the RSSS data are aggregated over a field of up to 10 ha. Short‐range variability in available Mg will therefore make a larger contribution to the variation of the NSI data; the physical aggregation of cores over a larger area in the RSSS survey smooths this variation. Under our hypothesis, this difference in distributions means that it would be inadvisable simply to combine the two datasets, despite their very similar mean values on the log scale, and we would expect an LMCR in which the two datasets are modelled as separate coregionalized variables to be more satisfactory than a univariate model applied to the pooled data.

**Table 1:** Summary statistics for all datasets on the concentration in the topsoil of plant‐available Mg (Mg_av_) on original and natural log scales. Representative sample scheme (RSSS) full set comprises all first observations on each sampled field. The reduced set excludes those fields without a unique coordinate. National Soil Inventory (NSI)

Dataset	Variable	Mean	Median	Standard deviation	Skewness
RSSS, full set	Mg_av_, mg l^‐1^	130.3	97	105.8	2.41
	Mg_av_, log mg l^‐1^	4.62	4.57	0.70	0.17
RSSS, reduced set	Mg_av_, mg l^‐1^	131.5	98	106.7	2.42
	Mg_av_, log mg l^‐1^	4.63	4.58	0.69	0.22
NSI	Mg_av_, mg l^‐1^	141.1	98	137.6	2.98
	Mg_av_, log mg l^‐1^	4.62	4.60	0.80	0.18

### *Variation over time*

**Figure 3:**
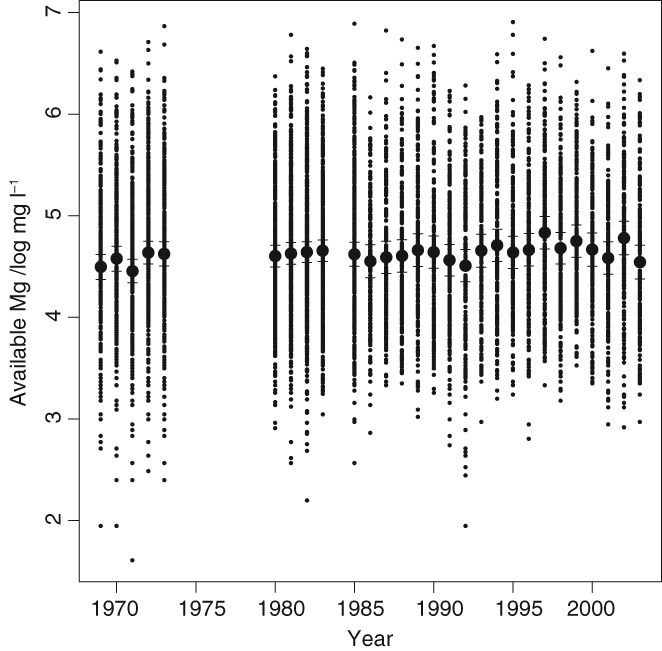
Concentrations of plant‐available Mg in the topsoil, transformed to natural logarithms, for sites in the RSSS dataset (first sample of each field only) plotted against year of sampling. The sample mean is shown by a large disc and the horizontal lines indicate the 95% confidence interval for the mean in each year.

Figure 3 shows the RSSS data (full data set, transformed to logarithms) plotted against time. The large discs show the annual mean values, and the horizontal bars show the bounds of the 95% confidence interval of the annual mean. There is no apparent systematic variation in available Mg over time. This is consistent with the findings of Baxter *et al*. (2006), who examined the RSSS data for four separate years from 1971 to 2001. Baxter *et al*. (2006) concluded that the spatial variation of available Mg in all years is dominated by differences between parent materials, which explains the temporal stability. Table 2 gives the anova for the available Mg with years as fixed effects. There is little difference between the between‐year mean square and the error (farms within years) mean square, and the null hypothesis of no difference between the years cannot be rejected (*P* = 0.196). Note that the between‐farms variance component is more than twice the within‐farms component, giving an intra‐farm correlation of 0.72. On this basis it was decided that all the RSSS data could be considered as a single set, pooling all times.

**Table 2:** Analysis of variance for effects of year on Mg, transformed to natural logarithms, in RSSS

Source	Degrees of freedom	Sum of squares	Mean square	Variance ratio	*P*‐value
Year	27	49.87	1.847	1.226	0.196
Error (farms within years)	2174	3274.6	1.506	—	—
Error (fields within farms	6486	879.5	0.136	—	—

Variance components	
Source	Variance
Between‐farms	0.350
Between‐fields within farms	0.136
Intra‐farm correlation	0.72

### *Validation and comparison of cokriging and univariate kriging predictions*

**Figure 4:**
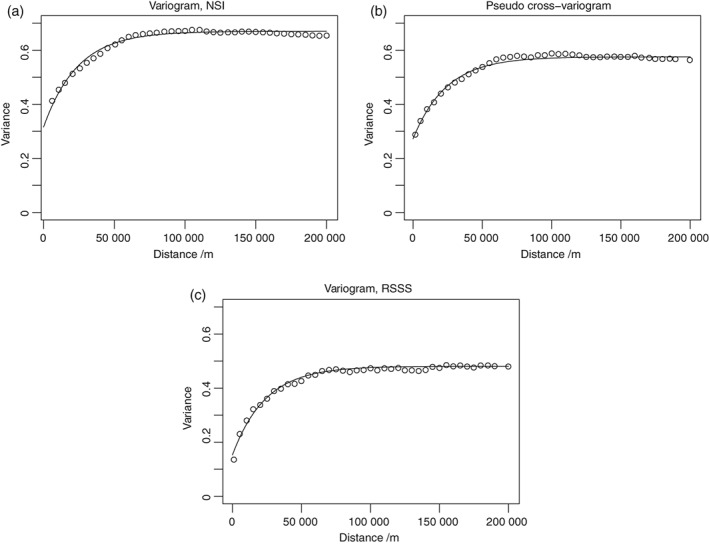
Point estimates of the auto‐variogram for available Mg, transformed to natural logarithms, from (a) the NSI data, (c) the RSSS data and (b) their pseudo cross‐variogram. The lines show the values of these functions according to the fitted LMCR parameters in Table 3(a).

Figure 4 shows the estimates of the auto‐ and pseudo cross‐variogram for log‐transformed available Mg in the RSSS and NSI datasets. The lines correspond to the LMCR with parameters listed in Table 3a.

**Table 3:** Parameters of (a) the linear model of coregionalization and (b) the variogram model for the combined dataset. Variances and covariances are in units of (log mg l^−1^)^2^

(a)	Linear model of coregionalization	
Nugget variance		
Coregionalization matrix		
	NSI	RSSS
NSI	0.316	0
RSSS		0.153
Structural correlation	0 (fixed)	

Correlated component	Exponential, a *a* = 24 029 m	
Coregionalization matrix		
	NSI	RSSS
NSI	0.354	0.303
RSSS		0.328
Structural correlation	0.89	

(b)	Variogram of combined RSSS and NSI data	
Nugget variance	0.245	
Correlated variance	0.322	
Distance parameter^a^, *a*	24 082 m	

a Parameter of the exponential variogram function *g*(*h*) = 1 − exp {−*h*/*a*}.

It is interesting to note that the major difference between the two datasets in the model is in the nugget variance component, the variance attributable to factors that vary over short distances not resolved spatially by the sampling. The nugget variance for the NSI data is more than twice that for the RSSS data. Again, this can be attributed to the smoothing effect of aggregation over the larger spatial support for RSSS. The LMCR shows spatial dependence in available Mg content up to a distance of about 80 km. The correlated variance components are very similar for the two datasets, and the structural correlation between them is strong (0.89), reflecting the common sources of variation contributing to both datasets.

**Figure 5:**
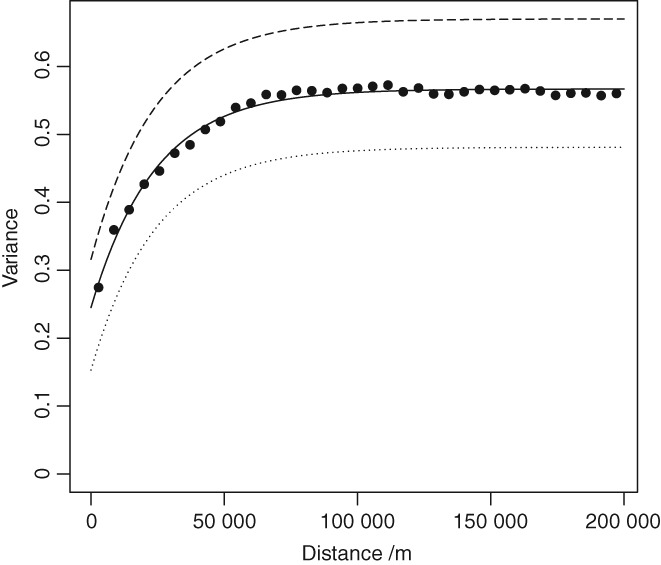
Point estimates of the auto‐variogram for available Mg, transformed to natural logarithms, from the combined NSI and RSSS dataset with the fitted model with parameters in Table 3(b). The dotted line shows the auto‐variogram of log‐transformed available Mg from the RSSS data and the dashed line the autovariogram for the NSI data according to the fitted LMCR parameters in Table 3(a).

Figure 5 shows the point estimates of the variogram estimated from the combined RSSS and NSI data, and a fitted model with parameters in Table 3(b). Additional lines on the plot show the autovariograms for the separate RSSS and NSI data according to the LMCR. As would be expected, the pooled variogram lies between the two autovariograms from the LMCR. The distance parameters are very similar but the effect of the different supports of the NSI and RSSS datasets on the variances is apparent.

**Figure 6:**
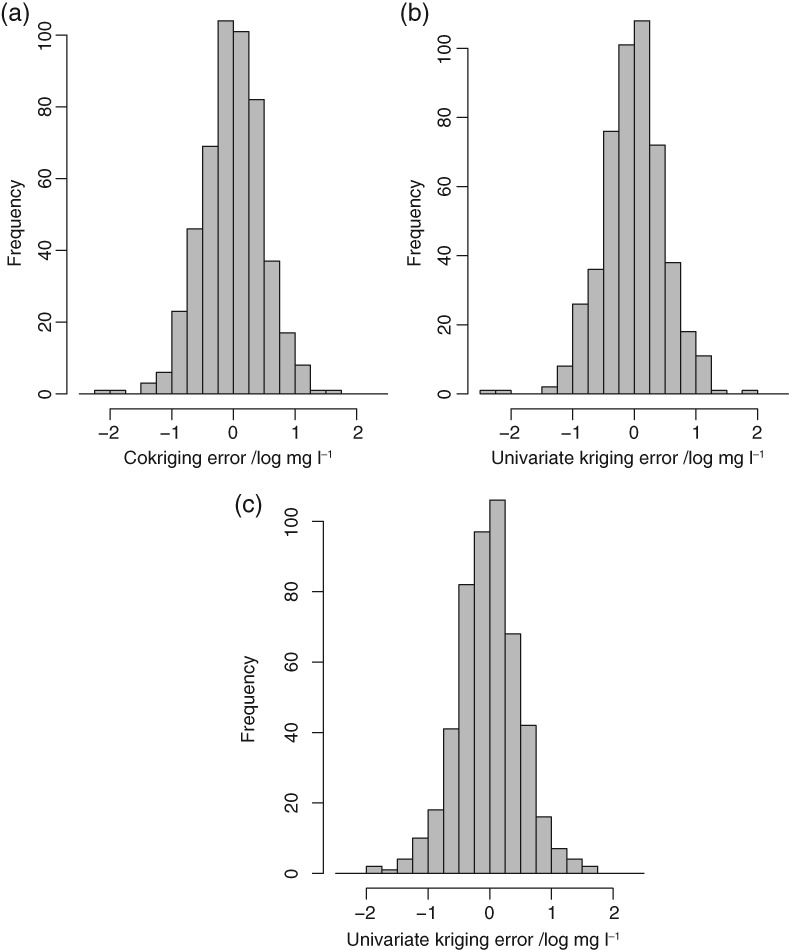
Histogram of (a) cokriging errors, (b) univariate ordinary kriging errors (RSSS data only) and (c) univariate ordinary kriging errors (combined NIS and RSSS data) at the 500 cross‐validation locations.

Figure 6 shows the histograms of the cross‐validation errors for the RSSS data by (6a) cokriging and (6b) univariate ordinary kriging from the RSSS data and (6c) univariate ordinary kriging from the combined RSSS and NSI data. The errors appear normally distributed, possibly with very few outlying values. The summary statistics for the cross‐validation (Table 4) show very small mean and median errors in all cases, reflecting the lack of bias of the kriging predictor. The mean square prediction error is largest for univariate kriging from the pooled RSSS and NSI data, and smallest for cokriging. The differences are small.

**Table 4:** Summary statistics for cross‐validation errors

Method	Variable	Units	Mean	Median
Ordinary cokriging	Prediction error	log mg l^‐1^	−0.02	−0.01
	Squared prediction error	(log mg l^‐1^)^2^	0.24	—
	Standardized squared	None	1.09	0.41
	prediction error			
Univariate ordinary kriging	Prediction error	log mg l^‐1^	−0.01	0
RSSS data only	Squared prediction error	(logmgl^‐1^)^2^	0.26	—
	Standardized squared	None	1.07	0.40
	prediction error			
Univariate ordinary kriging	Prediction error	log mg l^‐1^	−0.02	0.01
Combined NSI and RSSS data	Squared prediction error	(log mg l^‐1^)^2^	0.27	—
	Standardized squared	None	0.87	0.36
	prediction error			

**Figure 7:**
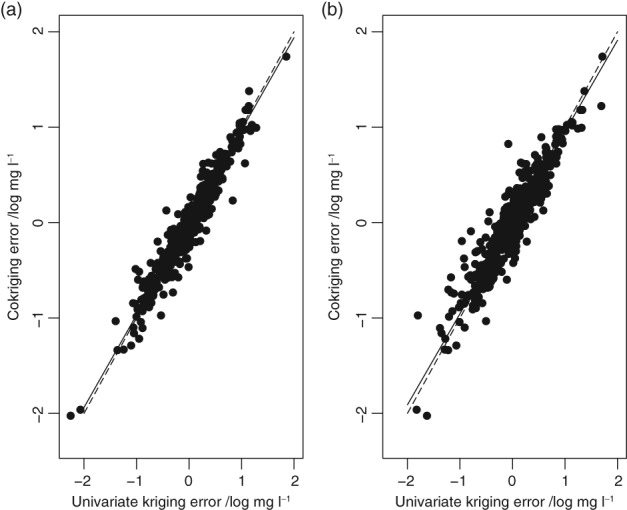
Scatterplot of univariate ordinary kriging error against cokriging error at the 500 cross‐validation locations for (a) univariate kriging from the RSSS data only and (b) univariate kriging from the combined NSI and RSSS data. The dashed line is the bisector and the solid line is the major axis of the dataset. Units are mg l^‐1^ of plant‐available soil Mg, transformed to natural logarithms.

The kriging cross‐validation errors for univariate kriging (RSSS and pooled data sets) are plotted against the cokriging errors in Figure 7. The major axis of each plot was computed using the smatr package in R (Warton *et al.,* 2012). In both cases the slope of the major axis is significantly smaller than 1.0 (0.969, *P* = 0.012 for ordinary kriging from the RSSS data only; 0.955, *P* = 0.004 for ordinary kriging from the combined NSI and RSSS data) as judged by the test of Pitman (1939). Thus, although the differences in the mean squared prediction errors are small, the variance of the cokriging errors is statistically significantly smaller than in either case of univariate ordinary kriging.

Note that the mean standardized squared prediction error is close to 1, and that the median value is close to the expected value in the case of unbiased kriging variances (0.45, with a 95% confidence interval for a sample of 500 of 0.36–0.55) for both cokriging and univariate kriging from the RSSS data alone. However, for univariate kriging from the combined RSSS and NSI data, the median square prediction error is on the lower bound of this confidence interval (0.36). This suggests that the kriging variance tends to overestimate the prediction error variance. This can be attributed to the effect on the variogram of adding the NSI data. The variances are increased.

**Figure 8:**
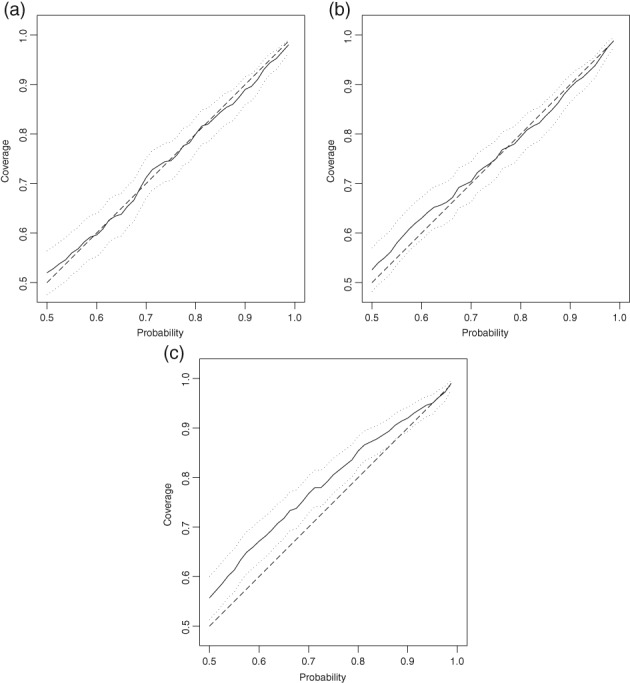
Plot of estimated coverage of prediction intervals for probabilities from 0.5 to 0.99 at the cross‐validation locations with their 95% confidence interval (dotted lines). The dashed line is the bisector. Prediction intervals are for (a) cokriging with the LMCR in Table 3(a), (b) univariate ordinary kriging from the RSSS data only with the auto‐variogram model in Table 3(a) and (c) univariate ordinary kriging from the combined RSSS and NSI data with the variogram model in Table 3(b).

Figure 8 shows the coverages of the prediction intervals for different probabilities at the cross‐validation sites for each set of kriging predictions. It is notable that, over much of the range of probabilities, the 95% confidence interval for the coverage of the univariate ordinary kriging predictions from the combined RSSS and NSI datasets is above the bisector, indicating that the confidence intervals are too wide. This is consistent with the standardized squared prediction errors, suggesting that uncertainty of the kriging predictions on the RSSS support are not well characterized by kriging variances from the combined dataset. In contrast, the coverages for the cokriging prediction intervals sit consistently over the bisector, which is within the 95% confidence interval. The 95% confidence interval for the coverage of the prediction intervals from univariate ordinary kriging from the RSSS data alone includes the bisector over the whole range.

**Figure 9:**
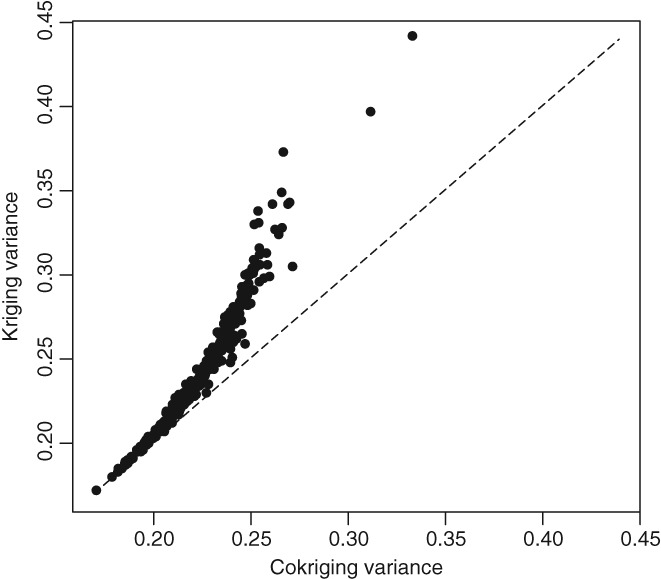
Plot of cokriging variance against univariate kriging variance for RSSS available Mg, transformed to natural logarithms, at the 500 cross‐validation locations. The dashed line is the bisector.

Figure 9 shows a plot of the cokriging variance against the univariate kriging variance. This shows that, although at many of the locations both these values are relatively small, the univariate kriging variance can be markedly larger because of the additional information provided by inclusion of the NSI data in the cokriging prediction.

### *Spatial variation of available Mg as mapped by cokriging*

**Figure 10:**
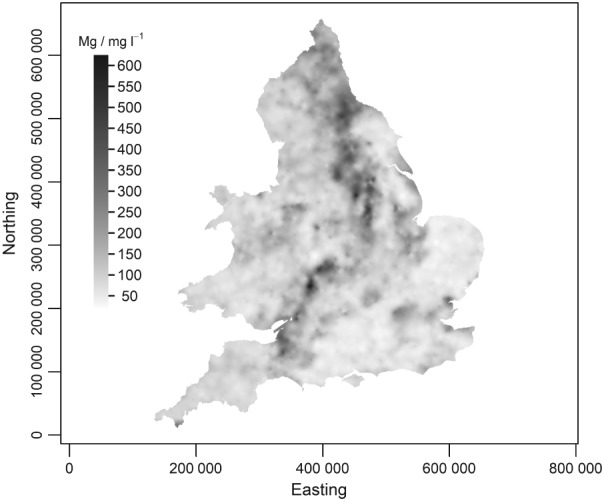
Median‐unbiased predicted concentrations of available Mg across England and Wales. Coordinates are in metres relative to the origin of the British National Grid.

**Figure 11:**
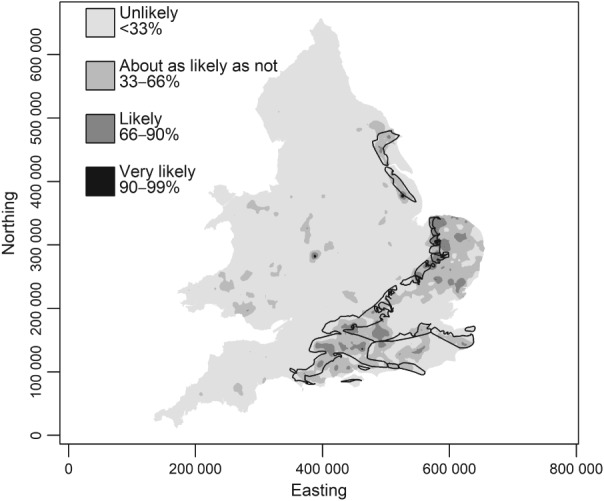
Probability, across England and Wales, that measured available Mg (RSSS support) would indicate that the soil Mg index is less than 2. The four legend units are defined verbally, and by a range of probability values, expressed as percentages. The polygons on the map show where the Chalk (Cretaceous) is present in outcrop based on British Geological Survey geological mapping at 1:50 000 ©NERC, with a cartographic simplification applied for visualization purposes. Coordinates are in metres relative to the origin of the British National Grid.

The cokriging predictions are on the log‐transformed scale. To obtain best linear unbiased predictions on the original scale requires a more complex back‐transformation than exponentiation. However, the exponentiation of the prediction, on the assumption of normal kriging errors (which is reasonable given Figure 6) gives a median‐unbiased prediction, and it can be argued that this is the more useful prediction for a markedly skewed variable (Pawlowsky‐Glahn & Olea, 2004). The median‐unbiased predicted values of available Mg (RSSS variable) are shown in Figure 10. On the basis of normal kriging errors one may compute, from the cokriging prediction and its variance, the probability at each prediction location that a measurement of soil Mg there would be ≤ 50 mg l^−1^ (i.e. below index 2 in the UK RB209 fertilizer recommendation system) (Defra, 2010; AHDB, 2017). According to the guidelines in AHDB (2017) a response to Mg is expected for arable crops on soil with an Mg index less than 2, and on grassland it is recommended to maintain available Mg at index 2 to avoid hypomagnesaemia (grass staggers) in livestock, although other factors contribute to the risk of this problem, notably excess available potassium. These probabilities are presented in Figure 11. Note that we have used calibrated verbal phrases based on Mastrandrea *et al*. (2010) for the plot legend. All probabilities ≤ 0.33 are termed ‘unlikely’. Values in the interval 0.33 < *x* ≤ 0.66 are termed ‘as likely as not’. The range of probabilities larger than 0.66 is divided into ‘likely’ and ‘very likely’ at 0.9. Following Lark *et al*. (2014), the map legend presents the probabilities (as percentages) together with the verbal phrases, to avoid some of the problems that have been shown to occur with purely verbal expressions (Budescu *et al*., 2009).

The distribution of available Mg shown in Figure 10 is the most detailed map of that variable published to date, because it uses all the RSSS data and NSI data (by cokriging). Previous maps used either just the NSI data (McGrath & Loveland, 1992) or subsets of the NSI data (Baxter *et al*., 2006). The dominant features of the map include the large available Mg concentrations associated with a north‐south band from the north‐east coast (near {443 000,540 000} on Figure 10) to the East Midlands (near {460 000,345 000} on Figure 10). This reflects the influence of Permo‐Triassic geology on the soil, notably the Magnesian Limestone (Permian), which contains dolomite (CaMg(CO_3_)_2_) (Stone *et al*., 2010). This is parent material for the soil *in situ* but Stone *et al*. (2010) also note that significant amounts are extracted for use as agricultural lime. There are also larger soil Mg concentrations in the south‐west midlands (near {410 000,264 000} on Figure 10), extending to the Avon region (near {360 000,190 000}), also over Triassic bedrock geology. These features are also seen on the map of total soil Mg presented by Rawlins *et al*. (2012), which also shows large concentrations in the Somerset Levels region (near {337 000,136 000}), which might be related to Quaternary marine incursions. It is instructive to note, however, that the large total Mg concentrations that Rawlins *et al*. (2012) showed over the Devonian Old Red Sandstone in the Welsh Borders area (near {333 000,258 000}) are not reflected in the map of available Mg. Similarly, although concentrations of available Mg are larger in the East Anglian Fens (near {554 000,330 000}) and on the Lincolnshire coast to the north than in surrounding soils, they are smaller here than over the Permo‐Triassic parent materials mentioned above. The total Mg concentrations mapped by Rawlins *et al*. (2012) in these regions are comparable. This highlights the need for caution when interpreting geochemical maps based on total element concentrations when one's primary interest is in agronomic problems.

**Figure 12:**
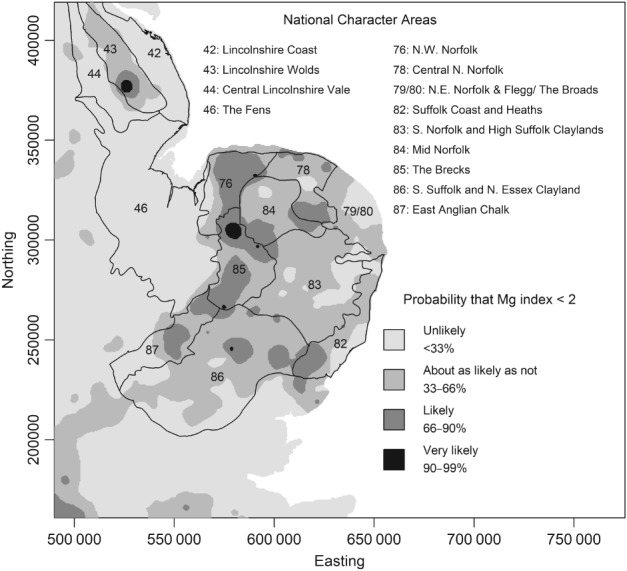
Probability within part of the East of England that measured available Mg (RSSS support) would indicate that the soil Mg index is less than 2. The four legend units are defined verbally, and by a range of probability values, expressed as percentages. The polygons on the map show National Character Areas according to the Natural England (2014) classification. The numbers in the polygons are those used by Natural England (2014) and the names of each National Character Area are shown on the legend. Used under Open Government Licence v3.0. Coordinates are in metres relative to the origin of the British National Grid.

Small concentrations of Mg are most notable in parts of north‐east and eastern England and East Anglia and in parts of the south of England, but to interpret these we focus on Figure 12. Figure 12 shows the probability that the observed soil Mg index at a site would be less than 2 at a site. Recall that these probabilities are at the RSSS support of a field or subregion of a field up to 10 ha, which is an appropriate support on which to interpret the risk that a plant nutrient limitation might be expressed. Superimposed on this figure is a generalized representation of the outcrop of the Chalk (Cretaceous). Note that much of the mapped area where it is judged to be ‘likely’ or ‘very likely’ that soil Mg is below index 2 is over the Chalk outcrop. The Mg content of the Chalk is small, and it is over such parent material, or very acid leached soils, that small concentrations of available Mg are typically expected (Simpson, 1983). Deficiency may be increased where soil is limed with material with small concentrations of Mg relative to Ca.

We look in more detail at one part of England where many of the sites where Mg deficiency is ‘likely’ or ‘very likely’ are found. This is East Anglia and east Lincolnshire (Figure 12). One reason to focus on this area is its importance in UK agriculture. The Eastern region of England has the largest total crop output of all regions in the country, and the largest total income from farming (Defra, 2016). In Figure 12 we show boundaries of the National Character Areas (NCA) used by the UK Government agency Natural England (Natural England, 2014). The area of England is divided into 159 such areas, which are defined with respect to geology and landscape and associated ecological and economic characteristics. They therefore reflect both parent material and anthropogenic influences on the soil. The NCAs are used as a basis for a range of policy decisions (notably on planning), the development of land management plans, including stewardship plans to encourage environmentally sensitive farming, and minerals planning.

In East Anglia and on the Lincolnshire Coast (Figure 12) there are clear relationships between the spatial pattern of risk of Mg deficiency and the NCAs. The Lincolnshire Wolds NCA (43) contrasts markedly with the neighbouring areas (42 and 44, the Lincolnshire Coast and Central Lincolnshire Vale, respectively). The Wolds NCA (43) overlies chalk, limestone, ironstone and clays and the soils are typically shallow and calcareous (Natural England, 2014). Within almost all the mapped area in this NCA the probability that the Mg index is less than 2 is larger than 33%, and there is an area where a soil Mg index less than 2 is ‘likely’ or ‘very likely’. The soil parent material in NCAs 42 and 44 comprises Quaternary sands and gravels over Chalk, and coastal sediments (Webster & Oliver, 2007), and glacial till over Jurassic mudstones respectively. Over the mapped area in these two NCAs the probability that the soil Mg index is less than 2 is deemed ‘unlikely’ (< 33%) almost everywhere. Here parent material is clearly a factor that controls the risk of deficiency. It is notable that arable land use dominates NCA 43, and growers here should be aware of the risks of limitations through small Mg concentrations.

It is ‘unlikely’ that a site in NCA 46, the Fens, is deficient in Mg. This is unsurprising as much of the soil in this area is derived from organic matter and marine deposits from the Quaternary, subject to artificial drainage since the seventeenth century (Godwin, 1978). The Chalk underlies the mapped area to the east of NCA 46, but with variable superficial cover. The three westernmost NCAs south or east of the Fens are 87 (East Anglian Chalk), 85 (The Brecks) and 76 (North West Norfolk). Arable agriculture predominates in these three NCAs, although there is substantial land under forestry in the Brecks (85) and some mixed farming in North West Norfolk (76). Much of the area where available Mg, interpreted at the RSSS support, is likely or very likely to be less than index 2 falls in these three NCAs. NCA 87 has shallow soils formed in blown sand over the Chalk dipslope. The underlying Chalk therefore has a strong influence on the soil and the overlying light‐textured parent material is subject to leaching. The Brecks (NCA 85) have soils largely formed in sand over the Middle and Upper Chalk. These soils were subject to considerable periglacial perturbation during the last ice age so that soil reaction varies markedly from calcareous to acidic over short distances. The soils are very free draining and ‘Breckland’ soils have a reputation for poor fertility (Curtis *et al*., 1976), the name reflecting how, historically, much land in the area was ‘breck’, or in fallow, either to restore fertility or because the prices of agricultural products were low. Figure 12 indicates that inadequate available Mg is an important component of nutrient limitation in these soils. The North West Norfolk NCA (76) also comprises sandy soils over Chalk. These are generally regarded as fertile and versatile soils, particularly in the east of the area, referred to as the ‘Good Sands’ (Natural England, 2014). However, at sites over most of this NCA it is ‘likely’ that the soil Mg index is less than 2 (Figure 12), and growers and their advisors in this area should be aware of this risk.

Further to the east the remaining seven NCAs are predominantly over sites where the probability that the Mg soil index is less than 2 is ‘about as likely as not’ (i.e. between 33 and 66%). However, there are some patches where the probability is larger. One such NCA is Mid Norfolk (NCA 84). The soils here show complex spatial variation reflecting a mixture of superficial materials (gravels, sand and glacial till) over the underlying Chalk. Central North Norfolk (NCA 78) shows a similar complexity for the same reasons. However, in contrast, NCA 83 (South Norfolk and High Suffolk Claylands) includes no significant area where the probability of an Mg index less than 2 is deemed ‘likely’ or ‘very likely’. The soils here are also formed in superficial material over the Chalk. The area is largely a flat plateau and on this plateau the soils are heavy clay, with some influence of the underlying Chalk. In the north east of the region is NCA 79 (North East Norfolk and Flegg) along with The Broads (NCA 80, artifical waterways (flooded peat workings) and three major tidal rivers). The soils of NCA 79 are deep and fertile, formed in Pleistocene Crag deposits and some overlying glacial till. Nowhere in NCA 79 and 80 is it deemed more than ‘as likely as not’ that the soil Mg index is less than 2.

The South Suffolk and North Essex Clayland (NCA 86) is a gently undulating plateau in chalky boulder clay (glacial till) over the Chalk. In places the soil is influenced by chalk fragments, and this may be reflected in the presence of some areas where the probability that soil Mg is less than index 2 exceeds 66%. The Suffolk Coast and Heaths NCA (82) comprises soils formed primarily in sand, gravel and glacial till over Pleistocene Crag deposits. Note that the probability that soil Mg is less than index 2 exceeds 66% over part of this NCA in the south and west, near the chalky boulder clay of NCA 86.

## Discussion

These analyses show how multivariate geostatistics can be used to illuminate how datasets on a soil property differ from each other and how they can be combined for improved spatial prediction.

*A priori*, we had reason to expect differences in the variability of available Mg from the difference in sample support, although the sample depth and analytical methods were the same. This was reflected in the simple summary statistics of the data. The geostatistical analysis provided further insight. It was notable that the nugget variance components for the NSI and RSSS datasets in the fitted LMCR were markedly different, with the former dataset having a much larger nugget. This is consistent with the difference in sample supports, because the nugget variance is the variance contributed by factors that occur at distances too short to be resolved by the sampling. It is variation at these scales that will be most markedly reduced by the aggregation of soil material over a region up to 10 ha in the RSSS.

Despite this difference in nugget variance, the spatially correlated variances of the two datasets were similar. This reflects the common factors influencing variation in available Mg at distances up to tens of kilometres. Although the RSSS and NSI data are not colocated, the LMCR allows us to show that the spatially correlated components of the two datasets are strongly correlated (structural correlation of 0.89).

The cokriging procedure then allowed us to make predictions of available Mg, treating the RSSS data as the primary variable, and using the NSI data to improve predictions. The cross‐validation showed that the mean squared errors of prediction are only slightly improved by including the extra data, although the tests on the slope of the major axis of the plot of cross‐validation errors for cokriging against ordinary kriging both from the RSSS data alone and from the pooled RSSS and NSI data showed that the variance of prediction errors was statistically significantly smaller for cokriging. The fact that addition of the NSI data to prediction by cokriging had only a small effect on the validation errors at RSSS sites might be surprising. Howevever, this can be explained by the spatial distribution of RSSS sites. Whereas the NSI data points are on a regular grid, the RSSS points are selected by probability sampling from land in agricultural use. If the 1713 farms in the dataset we used were distributed on a square grid over the sampled area, the grid spacing would be close to 9 km. In fact, the mean distance between a farm in the sample and its nearest neighbour in the sample is 4.6 km, and the median distance is 4.1 km. This indicates that the sample points show a distinctly clustered distribution. The addition of the NSI data, on a 5‐km grid, is likely to have a relatively small effect on the average density of observations in the vicinity of a cross‐validation point from the RSSS data, and hence on the precision of the predicted value by cokriging. However, the plot of cokriging and kriging variances (Figure 6) shows how cokriging limits the larger prediction error variances that may occur where the data on the primary variable are sparser.

Both the median standardized square prediction errors and the coverages of prediction intervals on the cross‐validation data show that the LMCR provides a valid model of the joint variation of the NSI and RSSS data, and allows us to predict Mg content on the RSSS support and to have confidence in the cokriging variances as measures of prediction uncertainty. The same holds for ordinary kriging prediction from the RSSS data alone. However, when the RSSS and NSI data were pooled, the standardized square prediction errors and coverages of the prediction intervals for cross‐validation of the variogram model by ordinary kriging showed that the model does not appear to reflect the behaviour of a stationary Gaussian random variable. This can be attributed to the difference in support between the two datasets, which means that they do not have a uniform variance. The predictions at the RSSS validation site, although showing no evidence of bias, have kriging variances that overestimate their uncertainty, and the coverages of the prediction intervals are therefore generally larger than the specified probability. This supports our initial hypothesis that the two variables cannot be combined simply, because of the difference in their support, despite the very similar mean values. Our results show that the LMCR is a useful statistical model for combining two measurements of the same variable that cannot be treated as having homogeneous variances.

Although the LMCR offers a method to combine two datasets on the same variable, which differ in their support and hence their variance, we note that the model could be used in even more general conditions. If, for example, the NSI data had been obtained with a different analytical method and so also differed from the RSSS data in their mean, or differed in their mean because they were measured over a different depth interval, it would still be possible to use the LMCR to apply the two datasets together to predict the target soil variable according to the specification for either (or both) of the datasets. In this case study we concluded that the RSSS data showed no evidence of a temporal trend in available Mg, and we therefore treated the whole dataset as a single variable. If this had not been the case then an alternative approach would have been to subdivide the RSSS data into smaller subsets over shorter time intervals, and to treat these, with the NSI data, as coregionalized random variables with an LMCR.

The map of available Mg in soil produced by cokriging (Figure 10), and the map showing the probability that the soil Mg index is less than 2 (Figure 11), show national‐scale variations with broad patterns that express underlying bedrock and superficial geology. The probability that the soil Mg index is less than 2 can be examined in more detail in the agriculturally intensive Eastern region (Figure 12), and can be related to the National Character Areas (Natural England, 2014) here. National Character Areas over which an Mg index less than 2 on the RSSS support is ‘unlikely’ include the Fens, the Lincolnshire Coast and the Central Lincolnshire Vale. Such a limitation is ‘likely’ or ‘very likely’ over much of North West Norfolk, The Brecks and the East Anglian Chalk, and this might be a localized risk in parts of the South Suffolk and North‐Essex Clayland, the south of the Suffolk Coast and Heaths, Mid Norfolk and North West Norfolk. A soil Mg index less than 2 is ‘as likely as not’ or ‘unlikely’ in the South Norfolk and High Suffolk Claylands, North East Norfolk and The Broads and on most of the Suffolk Coast and Heaths. The general pattern in this region is dominated by the bedrock geology (larger risks over the Chalk and smallest over the Pleistocene Crag or Fenland deposits) and influenced locally by the superficial material and its heterogeneity in composition and thickness. The geostatistical mapping of the probability of a small Mg index on the RSSS support facilitates this interpretation.

## Conclusions

To conclude, the RSSS and NSI national‐scale data on available Mg can be modelled as linearly coregionalized random variables. This model shows how the difference in sample support affects the short‐range variation of the data, and shows the strong relationship between the spatially correlated variation. The model then permits the two datasets to be combined for purposes of spatial mapping of available Mg, and cross‐validation shows that the uncertainty of the predictions is quantified reliably. If we disregard the differences in support between the two datasets and simply combine them into one then the uncertainty of the predictions cannot be quantified with confidence. This approach is of general interest because it illustrates how different datasets, collected for different purposes with different methodologies, can be combined in a statistically sound way.

## References

[ejss12743-bib-0001] AHDB (Agriculture and Horticulture Development Board) 2017 Nutrient Management Guide (RB209). Section 1 Principles of Nutrient Management and Fertiliser Use. AHDB, Stoneleigh [WWW document] URL https://ahdb.org.uk/documents/RB209/RB209_Section1_WEB_2017‐12‐01.pdf [accessed on 26 October 2018].

[ejss12743-bib-0002] Baxter, S.J. , Oliver, M.A. & Archer, J.R. 2006 The Representative Soil Sampling Scheme of England and Wales: the spatial variation of topsoil nutrient status between 1971 and 2001. Soil Use and Management, 22, 383–392.

[ejss12743-bib-0003] Blaker, H. 2000 Confidence curves and improved exact confidence intervals for discrete distributions. Canadian Journal of Statistics, 28, 783–798.

[ejss12743-bib-0004] Budescu, D.V. , Broomell, S. & Por, H.‐H. 2009 Improving communication of uncertainty in the reports of the Intergovernmental Panel on Climate Change. Psychological Science, 20, 299–308. 19207697 10.1111/j.1467-9280.2009.02284.x

[ejss12743-bib-0005] Chilès, J.‐P. & Delfiner, P. 2012 Geostatistics: Modeling Spatial Uncertainty, 2nd edn. John Wiley & Sons, Hoboken, NJ.

[ejss12743-bib-0007] Church, B.M. & Skinner, R.J. 1986 The pH and nutrient status of agricultural soils in England and Wales, 1969–1983. Journal of Agricultural Science, 107, 21–28.

[ejss12743-bib-0008] Clark, I. , Basinger, K.L. & Harper, W.V. 1989 MUCK—a novel approach to cokriging In: Proceedings of the Conference on Geostatistical, Sensitivity and Uncertainty Methods for Ground‐Water Flow and Radionuclide Transport Modeling (ed. Buxton B.E.), pp. 473–493. Battelle Press, Columbus, OH.

[ejss12743-bib-0009] Curtis, L.F. , Trudgill, S.T. & Courtney, F.M. 1976 Soils in the British Isles. Longman, London.

[ejss12743-bib-0010] Defra (Department for Environment, Food and Rural Affairs) 2010 Fertiliser Manual (RB209). HMSO, London [WWW document]. URL https://ahdb.org.uk/documents/rb209‐fertiliser‐manual‐110412.pdf [accessed on 26 October 2018].

[ejss12743-bib-0011] Defra (Department for Environment, Food and Rural Affairs) 2016 Regional Level Aggregate Farm Accounts for England. National Statistics, London [WWW document]. URL https://assets.publishing.service.gov.uk/government/uploads/system/uploads/attachment_data/file/676313/agriaccounts_regstatsnotice‐25jan18.pdf [accessed on 26 October 2018].

[ejss12743-bib-0012] Deutsch, C.V. & Journel, A.G. 1998 GSLIB Geostatistical Software and User's Guide, 2nd edn. Oxford University Press, New York.

[ejss12743-bib-0013] Emmett, B.A. , Frogbrook, Z.L. , Chamberlain, P.M. , Griffiths, R. , Pickup, R. , Poskitt, J. *et al* 2008 *Soils manual*. Countryside Survey Technical Report no 03/07. Centre for Ecology and Hydrology, Lancaster.

[ejss12743-bib-0014] Freund, J.E. 1992 Mathematical Statistics, 5th edn. Prentice‐Hall, Englewood Cliffs, NJ.

[ejss12743-bib-0015] Godwin, H. 1978 Fenland: Its Ancient Past and Uncertain Future. Cambridge University Press, Cambridge, UK.

[ejss12743-bib-0016] Goovaerts, P. & Webster, R. 1994 Scale‐dependent correlation between topsoil copper and cobalt concentrations in Scotland. European Journal of Soil Science, 45, 79–95.

[ejss12743-bib-0017] Journel, A.G. & Huijbregts, C.J. 1978 Mining Geostatistics. Academic Press, London.

[ejss12743-bib-0018] Lagacherie, P. , Bailly, J.S. , Monestiez, P. & Gomez, C. 2011 Using scattered hyperspectral imagery data to map the soil properties of a region. European Journal of Soil Science, 63, 110–119.

[ejss12743-bib-0019] Lark, R.M. 2000 A comparison of some robust estimators of the variogram for use in soil survey. European Journal of Soil Science, 51, 137–157.

[ejss12743-bib-0020] Lark, R.M. 2002 Robust estimation of the pseudo cross‐variogram for cokriging soil properties. European Journal of Soil Science, 53, 253–270.

[ejss12743-bib-0021] Lark, R.M. 2012 Some considerations on aggregate sample supports for soil inventory and monitoring. European Journal of Soil Science, 63, 86–95.

[ejss12743-bib-0022] Lark, R.M. & Papritz, A. 2003 Fitting a linear model of coregionalization for soil properties using simulated annealing. Geoderma, 115, 245–260.

[ejss12743-bib-0023] Lark, R.M. , Bellamy, P.H. & Rawlins, B.G. 2006 Spatio‐temporal variability of some metal concentrations in the soil of eastern England, and implications for soil monitoring. Geoderma, 133, 363–379.

[ejss12743-bib-0024] Lark, R.M. , Ander, E.L. , Cave, M.R. , Knights, K.V. , Glennon, M.M. & Scanlon, R.P. 2014 Mapping trace element deficiency by cokriging from regional geochemical soil data: a case study on cobalt for grazing sheep in Ireland. Geoderma, 226–227, 64–78.

[ejss12743-bib-0025] MAFF (Ministry of Agriculture, Fisheries and Food) 1986 The Analysis of Agricultural Materials, 3rd edn. Reference Book 427. HMSO, London.

[ejss12743-bib-0026] Mastrandrea, M.D. , Field, C.B. , Stocker, T.F. , Edenhofer, O. , Ebi, K.L. , Frame, D.J. *et al* 2010 *Guidance Note for Lead Authors of the IPCC Fifth Assessment Report on Consistent Treatment of Uncertainties* Intergovernmental Panel on Climate Change (IPCC). [WWW document]. URL http://www.ipcc.ch/pdf/supporting‐material/uncertainty‐guidance‐note.pdf [accessed on 26 October 2018].

[ejss12743-bib-0027] McGrath, S.P. & Loveland, P.J. 1992 The Soil Geochemical Atlas of England and Wales. Blackie Academic and Professional, Glasgow.

[ejss12743-bib-0028] Myers, D.E. 1991 Pseudo‐cross variograms, positive‐definiteness and cokriging. Mathematical Geology, 23, 805–816.

[ejss12743-bib-0029] Natural England . 2014 *National Character Area Profiles: Data for local decision making* [WWW document]. URL https://www.gov.uk/government/publications/national‐character‐area‐profiles‐data‐for‐local‐decision‐making/national‐character‐area‐profiles [accessed on 26 October 2018].

[ejss12743-bib-0030] Papritz, A. & Webster, R. 1995 Estimating temporal change in soil monitoring: I. Statistical theory. European Journal of Soil Science, 46, 1–12.

[ejss12743-bib-0031] Papritz, A. , Künsch, H.R. & Webster, R. 1993 On the pseudo cross‐variogram. Mathematical Geology, 25, 1015–1026.

[ejss12743-bib-0032] Pawlowsky‐Glahn, V. & Olea, R.A. 2004 Geostatistical Analysis of Compositional Data. Oxford University Press, New York.

[ejss12743-bib-0033] Pitman, E.T.G. 1939 A note on normal correlation. Biometrika, 31, 9–12.

[ejss12743-bib-0034] R Core Team 2017 R: A Language and Environment for Statistical Computing. R Foundation for Statistical Computing, Vienna [WWW document]. URL https://www.R‐project.org/ [accessed on 26 October 2018].

[ejss12743-bib-0035] Rawlins, B.G. , McGrath, S.P. , Scheib, A.J. , Breward, N. , Cave, M. , Lister, T.R. *et al* 2012 The Advanced Soil Geochemical Atlas of England and Wales. British Geological Survey, Keyworth, Nottingham [WWW document]. URL https://www.bgs.ac.uk/gbase/advSoilAtlasEW.html [accessed on 26 October 2018].

[ejss12743-bib-0036] Rawlins, B.G. , Marchant, B. , Stevenson, S. & Wilmer, W. 2017 Are data collected to support farm management suitable for monitoring soil indicators at the national scale? European Journal of Soil Science, 68, 235–248.

[ejss12743-bib-0037] Robinson, N.J. , Benke, K.K. , Norng, S. , Kitching, M. & Crawford, D.M. 2017 Improving the information content in soil pH maps: a case study. European Journal of Soil Science, 68, 592–604.

[ejss12743-bib-0038] Scherer, R. 2018 *PropCIs: Various Confidence Interval Methods for Proportions* R package version 0.3‐0 [WWW document]. URL https://CRAN.R‐project.org/package=PropCIs [accessed on 26 October 2018].

[ejss12743-bib-0039] Simpson, K. 1983 Soil. Longman, London.

[ejss12743-bib-0040] SNIFFER 2007 National Soil Monitoring Network: Review and Assessment Study. SNIFFER, Edinburgh, LQ09, June 2007.

[ejss12743-bib-0041] Stone, P. , Millward, D. , Young, B. , Merritt, J.W. , Clarke, S.M. , McCormac, M. *et al* 2010 British Regional Geology: Northern England, 5th edn. British Geological Survey, Keyworth.

[ejss12743-bib-0042] Warton, D.I. , Duursma, R.A. , Falster, D.S. & Taskinen, S. 2012 smatr 3 – an R package for estimation and inference about allometric lines. Methods in Ecology and Evolution, 3, 257–259.

[ejss12743-bib-0043] Webster, R. & Oliver, M.A. 2007 Geostatistics for Environmental Scientists, 2nd edn. John Wiley, Sons, Chichester.

